# The clinical significance of emotional urgency in bipolar disorder: a scoping review

**DOI:** 10.1186/s40359-024-01700-1

**Published:** 2024-05-15

**Authors:** Wen Lin Teh, Sheng Yeow Si, Jianlin Liu, Mythily Subramaniam, Roger Ho

**Affiliations:** 1 Institute of Mental Health, 10 Buangkok View, Buangkok Green, Medical Park, Singapore, S539747 Singapore; 2 Yong Loo Lin School of Medicine, National University of Singapore, 21 Lower Kent Ridge Rd, Singapore, S119077 Singapore; 3 MOH Holdings, 1 Maritime Square, #11-25, Singapore, S099253 Singapore; 4 Institute of Health Innovation and Technology (iHealthtech), National University of Singapore, 14 Medical Dr, #14-01 MD6, Singapore, S117599 Singapore

**Keywords:** Emotion-based impulsivity, Emotional urgency, Bipolar disorder, UPPS

## Abstract

**Background:**

Emotional urgency, defined as a trait concept of emotion-based impulsivity, is at least moderately associated with general psychopathology. However, its clinical significance and associations with clinically relevant features of bipolar disorder remain unclear. This scoping review aims address this gap by determining the extent of evidence in this niche scope of study.

**Methods:**

Evidence of between-group differences of positive and negative urgency, its associations with mood severity, and all peripheral associations related to illness and psychosocial outcomes were synthesized based on PRISMA checklists and guidelines for scoping reviews (PRISMA-ScR).

**Design:**

Electronic databases were searched for articles published between January 2001 and January 2024. A total of 1013 entries were gathered, and a total of 10 articles were included in the final selection after the removal of duplicates and ineligible articles.

**Results:**

Differences in urgency scores between bipolar disorder and healthy controls were large (Cohen’s *d* ranged from 1.77 to 2.20). Negative urgency was at least moderately associated with overall trauma, emotional abuse, neglect, suicide ideation, neuroticism, and irritable/cyclothymic temperament, whereas positive urgency was at least moderately associated with various aspects of aggression and quality of life. Positive but not negative urgency was associated with quality of life in bipolar disorder.

**Conclusion:**

Large between-group differences found for emotional urgency in bipolar disorder imply large clinical significance. Emotional urgency was associated with worse clinical features and outcomes. Given the high clinical heterogeneity of the disorder, emotional urgency may be an important phenotype indicative of greater disorder severity.

## Introduction

Bipolar disorder (BD), which encompasses primarily bipolar I and II disorders, is a subcategory of mood disorders that is characterized by episodes of mania and depression causing significant dysfunction. A diagnosis of bipolar II disorder requires at least one depressive episode and a hypomanic episode, whereas a diagnosis of bipolar I requires only a manic episode [1, 2], though, research has shown that that the majority of individuals with bipolar I (94.2%) do report having experienced at least one depressive episode [3]. High mortality, disease burden, poor psychosocial functioning, and well-being, are several adverse outcomes associated with bipolar disorders [4–7].

Impulsivity is a core putative feature of bipolar disorders [8, 9] that becomes elevated during mania [10]. Impulsivity is typically conceptualized as the tendency to make rash decisions or responses that lead to undesirable consequences [11, 12]. The inability to inhibit undesired actions can adversely impact various aspects of functioning due to a disregard for future consequences in favour of immediate rewards [13–15]. Generally, trait (i.e., a predisposition toward rash actions) and behavioural facets (i.e., the lack of inhibition of ongoing actions, such as delay of gratification or response inhibition) of impulsivity are heightened in bipolar disorders [16–18]. According to meta-analytic studies, behavioural aspects are significantly impaired with medium effect sizes (Hedge’s *g* estimates ranging from 0.41 to 0.51; [18, 19]), whereas trait aspects, such as motor, cognitive, and non-planning impulsivity, commonly measured by the Barratt’s Impulsivity Scale (BIS), are significantly elevated in bipolar disorders [17, 20]. In addition, trait impulsivity has been associated with disorder onset [21], and certain sub-features have been linked prospectively to illness severity [22].

One facet of impulsivity that has gained popularity in recent decades is emotional urgency, defined as a trait-tendency to react impulsively amidst strong emotions. Emotional urgency represents a combination of the two dimensions (negative and positive urgency) of the UPPS-P Impulsive Behaviour scale (Urgency, Perseverance, Premeditation, Sensation Seeking, and Positive Urgency), which is an updated version of the original UPPS scale that features a total of five trait impulsivity dimensions: (lack of) premeditation, (lack of) perseverance, sensation seeking, negative and positive urgency [23]. Negative and positive urgency are referred to as trait tendencies of rash action amidst negative and positive emotions respectively—the latter dimension [24] is the most recent dimension incorporated into the UPPS-P, and it is also the least studied. Unlike other UPPS-P facets that are operationalized separately from emotionality, emotional urgency represents a unique aspect of impulsivity that ties rash decision making with intense positive and negative emotions [25, 26].

Each of the final five dimensions of the UPPS corresponds to a facet of the five-factor model of personality. For instance, negative urgency clusters closely with the personality trait factor neuroticism [12]. However, theories of emotional urgency have since moved beyond personality concepts due to a growth of neuroscience based research in cognition and emotion [27]. Emotional urgency is thus linked to impaired executive control and positively associated with maladaptive behaviours. Negative urgency has been associated with a neurocognitive vulnerability arising from reduced neurochemical activity or dysfunction in key neural areas of the ventromedial pre-frontal cortex and orbitofrontal cortex, which results in less efficacious regulatory control over pathological impulses (i.e., addictive impulses) from the basal ganglia and extended amygdala, and emotional arousal from sensory and visceromotor circuitries within the orbitofrontal cortex [28–30]. In an experimental study among non-clinical college students, it was found that only positive urgency (and not other facets of the UPPS-P) significantly and uniquely predicted risk-taking and increased alcohol consumption after a positive mood induction [31]. After facing (experimentally induced) social rejection, individuals with average to high levels of negative urgency were more likely than those with low levels to show increased impulsivity (i.e., failing to inhibit a prepotent response; [32]). In both experiments, non-emotional facets of impulsivity failed to achieve similar results as urgency, suggesting their relative smaller roles. Emotional urgency has been an important predictor of substance misuse (Smith and Cyders, 2016), gambling [33], drinking problems [34]. However, one may argue that based on conventional effect size cut-off criteria, the effect sizes are small (*r* =.23 for nicotine severity for instance; [35]).

Personality constructs typically do not yield large effect sizes — a medium effect size of .29, for instance, corresponds to the 75th percentile of all personality correlations; less than 3% of all personality correlations documented are large (*r* ≥.50; [36]). Thus, at the 75th percentile, personality constructs with correlations of *r* ≥.29 are considered to have large practical significance. A meta-analysis of 115 studies (*N* = 40,432) found that emotional urgency had the greatest effect on general psychopathology (a medium effect, *r* =.34) whereas non-emotional aspects of impulsivity only had a small effect (*r* ranging from .08 to .14). More crucially, the meta-analysis found large effects on depression (*r* =.45) and borderline personality disorder (*r* =.58), implying that negative urgency has greater relevance in disorders of negative mood dysregulation [37].

While a large repertoire of existing research has been dedicated to the study of general impulsivity in bipolar disorders, most have not examined emotional urgency. It is unclear if there is sufficient empirical evidence for its clinical significance. Systematic reviews published thus far have synthesized important work in non-emotional constructs of impulsivity [18, 20], impulsivity constructs in relation to addictions and substance misuse [26, 29, 30, 38–41], problematic eating and related disorders [26, 42, 43], aggression [44], self-injurious behaviours [45], psychosis with comorbid substance use [46], and general psychopathology [37]. However, to the best of knowledge, there have been no attempts to synthesize existing empirical evidence of positive *and* negative urgency in relation to important clinical and psychosocial factors in bipolar disorders.

Prevailing research shows that emotional urgency is more closely associated with psychopathology and externalizing behaviours (i.e., behaviours directed outwards or rule-breaking behaviours), such as outward aggression, gambling, substance use, than the remaining facets of the UPPS. Unlike the other facets, negative urgency is positively correlated with internalizing behaviours (i.e., behaviours that are inflicted inwards toward the self) with medium to large effect sizes, such as non-suicidal self-injurious behaviours (NSSI; d = 0.56 to 0.59, a medium effect size; [45, 47] and binge-eating (d = 0.64, a medium effect size; [42]. Emotional urgency underlies many forms of behavioural addictions [48, 49], risk-taking behaviours [18], and to a lesser extent, suicidality [50]. Furthermore, mood instability, irritability, depression, and mania, are part of spectrums of emotionality that, when heightened, can nudge individuals to engage in maladaptive behaviours [34, 37, 42, 45, 51]. While the association between negative emotions and maladaptive behaviours is well known and accepted in psychopathology, few studies have investigated the role of emotional urgency in this relationship. Finally, where mania is the primary mood state of concern, support for the association between emotional urgency and mania remains unclear.

Considering the existing gaps in research on emotional urgency in bipolar disorders, we conducted a scoping review to answer a fundamental question, “what is the clinical significance and clinically relevant correlates of emotional urgency in bipolar disorder?” This approach was adopted to determine the extent of evidence in a niche area of study before proceeding with a systematic review approach. Thus, this review aims to, firstly, determine the extent of emotional urgency’s clinical relevance by qualitatively summarizing prevailing research that reported between-group differences of emotional urgency scores (i.e. bipolar disorder vs. healthy controls, and/or vs. other clinical populations) and associations between emotional urgency and bipolar disorders (i.e. both categorical diagnosis and continuous symptom measures); and secondly, summarize clinically relevant associations between emotional urgency and all aspects relevant to illness (e.g., aetiological factors and clinical outcomes, psychiatric comorbidities), psychosocial outcomes (e.g., functioning or quality of life), and maladaptive behaviours (e.g., suicidality, self-harm) in individuals with bipolar disorders.

## Methods

### Protocol

The protocol was published in the International Prospective Register of Systematic Reviews (PROSPERO) on 2nd July 2021 (Reg no.: CRD42021258230) in preparation for a systematic review. However, a systematic synthesis of data did not materialize due to the small number of studies found. The quality of reporting and conduct of this scoping review is based on the Preferred Reporting Items for Systematic Reviews and Meta-Analyses (PRISMA) checklists and guidelines for scoping reviews (PRISMA-ScR; [52, 53]).

### Inclusion criteria

(1) Articles that investigated emotion-related impulsivity, emotional urgency, positive urgency, or negative urgency in bipolar disorders, its association (if any) with psychiatric comorbidities (i.e., anxiety disorders), and/or maladaptive behaviours (e.g., suicidality, self-harm); (2) work that had reported the use of at least one of the positive or negative urgency subscale of the UPPS/UPPS-P/PUM; (3) studies that had recruited adults who either met the Diagnostic Statistical Manual of Mental Disorders (DSM-IV or DSM-5) criteria for bipolar disorder or were recruited from a clinical setting; (4) peer-reviewed journal articles published between January 2001 and January 2024; and (5) written in the English language.

### Exclusion criteria

Conference abstracts, commentaries, editorials, reviews, meta-analyses, dissertations, qualitative studies, and case-series.

### Identification and selection of studies

Electronic databases, such as MEDLINE (PubMed), PsychINFO, Web of Science, and Embase, were searched to identify eligible articles published between January 2001 and January 2024. This comprised an initial search of articles between January 2001 and May 2023, and a secondary search conducted in February 2024 for articles published between June 2023 and January 2024, using the search syntax: (Bipolar, Mani* or cyclothymi* or manic-depressi* or hypomani*) AND (positive urgency or negative urgency or emotion* impuls* or emotion* urgency).

### Study selection

The primary reviewer (WLT) and a second reviewer (SYS) independently screened article titles and abstracts to determine study inclusion. Any discrepancies were resolved through consensus discussions; if consensus could not be reached, senior authors (JLL, MS, RCH) were consulted. Both the primary reviewer (WLT) and a second reviewer (SYS) further screened the articles independently based on full texts obtained and extracted the data.

### Data extraction and analysis

General information related to study characteristics including study design, recruitment setting, and sample size were extracted from each study. Emotional urgency and mood severity measures, clinical outcomes, group differences between bipolar disorders and various comparison groups (e.g., healthy controls), statistical associations between emotional urgency and bipolar disorder diagnosis (categorical), and mood symptoms were extracted to address the primary aim of the review. Any additional statistical associations between emotional urgency and illness (e.g., aetiological factors, clinical or recovery outcomes, psychiatric comorbidities), well-being (e.g., functioning or quality of life), or maladaptive behaviours (e.g., suicidality, self-harm) in individuals with bipolar disorders were extracted to address the secondary aim of the review. Given the small number of articles, an overall qualitative synthesis was deemed appropriate.

## Results

### Study selection

**Fig. 1:**
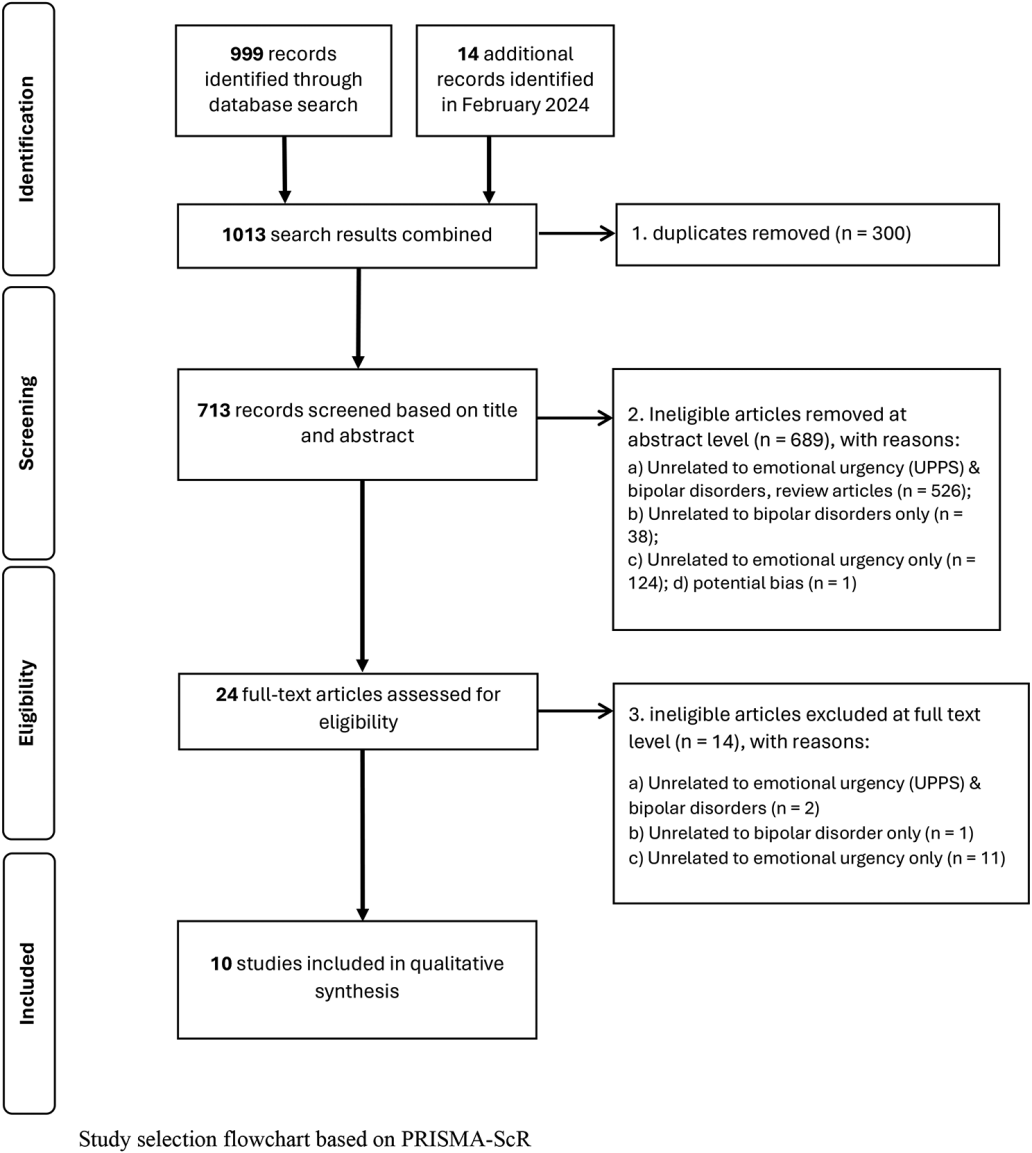
Flowchart for study selection flowchart based on PRISMA-ScR

The initial search yielded 999 entries. A total of 10 entries were included in the qualitative synthesis after the removal of 295 duplicates, 680 ineligible records (i.e., review articles, articles unrelated to emotional urgency and/or bipolar disorders) at the first screening at the abstract and title level, and 14 ineligible records after the second assessment at the full-text level (Bøen et al., 2015; Johnson et al., 2019, 2017; Johnson and Carver, 2016; Kwapil et al., 2000; Muhtadie et al., 2014; Quilty et al., 2010; Reich et al., 2019; Shakeel et al., 2019; Victor et al., 2011). An additional 14 entries were extracted from an additional search of publications between June 2023 and January 2024. However, none of the 14 additional entries were included for the following reasons: duplicates (5 entries), ineligible records (8 entries), and potential bias stemming from potential conflict of interest (1 entry is first author’s article). See Fig. 1 for the flowchart.

### Overview of included studies

In all 10 studies reviewed, clinical protocols (e.g., Structured Clinical Interview for DSM-IV) were used to assess participants’ diagnoses of bipolar disorder. Most studies reported a female majority. Only one study had explicitly indicated that participants were in the remission stage [54]. Seven out of ten of studies were conducted in the United States and the remaining three were conducted in Canada or Norway. All studies reviewed had relied on convenience sampling. Two studies [55, 56] had drawn data from a larger study [57] and may have overlapping datasets. Finally, most studies had investigated either positive or negative urgency; only three out of ten studies examined both dimensions in the same report. Three studies had used the original UPPS which comprises four factors of impulsivity by Whiteside and Lynam (2001), two studies had utilized the UPPS-Positive scale (UPPS-P) which comprises five factors of impulsivity (an additional factor of positive urgency), and five studies had adopted the Positive Urgency Measure (PUM) scale by Cyders et al. (2007). The UPPS-P, which contains components of the original UPPS and PUM, has been validated in psychiatric populations [58]. The total number of participants with bipolar disorders across all studies was 451. Sample sizes ranged from 19 to 145 participants, and study samples were composed of adult and young adult patients. Tables 1 and 2 summarizes between-group differences of emotional urgency scores and associations between emotional urgency and clinical/psychosocial outcomes respectively.

**Table 1:** Table of evidence of between-group differences of emotional urgency between bipolar disorders and the respective comparison groups; and association between mood severity and emotional urgency

Authors	Country	Diagnostic tool	Clinical Sample	Comparison Sample	Valence of Emotional Urgency	Emotional Urgency Measure	Mood Severity Measure	Between-Group Differences	Emotional Urgency-Mood Association
Quilty et al. (2010)	Canada	SCID	1. n: 137 (BP I & II) 2. Average age: 43.55 (11.64)	1. n: 138 (depressive dx) 2. Average age: 42.48 (11.54)	-ve	UPPS; α: 0.90 (negative urgency)	*nd*	1. bipolar dx: 32.06 (7.83) 2. depressive dx: 31.02 (8.03) 3. *t =* 1.05; *p* =.30	*nd*
Victor et al. (2011)	United States	SCID	1. n: 76 (BP I) 2. Average age: 37.12 (11.68) 3. 46 females	nil	+ve	PUM; α: *nd*	*nd*	1. BD I w/o substance use or alcohol dx 2. BD I w/ substance use or alcohol dx 3. *t* (68) = -2.51; *p* =.015 1. BD I w/o anxiety dx 2. BD I w anxiety dx 3. *t* (69) = -2.36; *p* =.021	
Muhtadie et al. (2014)	United States	SCID	1. n: 91 (BP I only; inter-episode period) 2. Average age: 37.8 (11.6) 3. 60% female 4. Average age of onset: 22 years (manic) or 18.2 years (depressive episode)	1. n: 80 2. Average age: 35 (12.1) 3. 52% female	-ve, +ve	PUM; α: 0.97 UPPS; α: 0.93 (negative urgency; sample 2)	1. BRMS; α: 0.77 2. modified MHRSD; α: 0.82 3. Hx of Bipolar Dx (Y/N)	Negative Urgency 1. BD: 3.64 (0.65) 2. HC: 2.92 (0.69) 3. *F =* 49.67; *p* <.001; η_p_^2^ = 0.226	Negative Urgency 1.Hx of Bipolar Dx; *b* = 0.31; *p* <.001; Δ*R*^*2*^ = 0.08; *p* <.001 2. BRMS: *ns* 3. modified HRSD: *ns* Positive Urgency 1. Hx of Bipolar Dx; *b* = 0.50; *p* <.001; Δ*R*^*2*^ = 0.2; *p* <.001 2. BRMS & MHRSD scores: *rs* <|0.17|, *ps >.11* Positive Urgency 1. BD: 33.74 (10.89) 2. HC: 21.00 (7.29) 3. F = 78.69; *p* <.001; η_p_^2^ = 0.316
Bøen et al. (2015)	Norway	MINI, SCID	1. n: 20 (BP II only) 2. Average age: 33.6(5.7) 3. 75.0% female 4. Average age of onset: 15.8(5.1)	1. n: 44 (healthy controls) 2. Average age: 28.8(7.7) 3. 77.8% female 1. n: 25 (Borderline PD) 2. Average age: 26.8 (6.1) 3. 88.0% females	-ve	UPPS	1. YMRS; α: *nd* 2. MDRAS; α: *nd*	1. BP II: 2.76(0.56) 2. HC: 1.70(0.39) 3. Borderline PD: 3.24 (0.52) 4. *F* = 95.3; *p* <.001; *d* = 2.20 BP II > HC: *p* <.001 BP II = Borderline PD: *p* =.003 BP II w/ high (*n* = 10; score ≥ 12) = low MADRAS (*n* = 10; score ≤ 11): *p =*.019	MADRAS and YMRS *p* >.17
Johnson et al. (2016)	United States	SCID	1. n: 58 (BP I in remission only) 2. Average age: 35.93 (12) 3. 54.2% female 4. Average age of onset: 21.22 (7.18)	*nd*	+ve	PUM; α: 0.97	1. YMRS; α: 0.82 2. MHRSD; α: 0.84	*nd*	YMRS: *r =*.02; *ns*, HMRSD: *r* =.30; *p* <.05
Johnson et al. (2017)	United States	SCID	1. n: 133 (BP I in remission only) 2. Average age: 36.81 (11.67) 3. 58.6% female 4. Age of onset: 21.85 (8.09)	1. *n*: 110 (healthy controls)	-ve, +ve	PUM; α: 0.97 UPPS; α: 0.93 (sample 2)	1. YMRS; α: *nd* 2. BRMS; α: 0.94 2. MHRSD; α: 0.84 (sample 1), α: 0.92 (sample 2)	Positive Urgency 1. BP (*n = 58; sample 1)*: 33.84 (9.55) 2. HC (*n* = 53; sample 1): 19.34 (6.01) 3. *t*(231)=-13.32; *p* <.001; *d =* 1.82	Negative Urgency MDD episode freq: *r* =.14; *ns* Positive Urgency MDD episode freq: *r* =.21; *p* <.05
Reich et al. (2019)	United States	SCID	1. n: 12 (BP I w/ suicide history) 2. Average age: 36.9 (14.9) 3. 4 females 1. n: 18 (BP I w/o suicide history) 2. Average a: 38.8 (11.7) 3. 5 females	1. n: 12 (healthy controls) 2. Average age: 33.1 (12.3) 3. 3 females	-ve, +ve	UPPS-P; α: *nd*	1. YMRS; α: *nd* 2. HAMD; α: *nd*	Negative Urgency 1. BP w suicide: 29.0 (8.2) 2. BP w/o suicide: 22.9 (8.5) 3. *t*(27) = 1.89; *p* =.07	
								Positive Urgency 1. BP w suicide: 35.5 (11.9) 2. BP w/o suicide: 26.6 (11.7) 3. *t*(27) = 1.99; *p* =.06	
Shakeel et al. (2019)	Canada	SCID-5	1. n: 19 (BD) 2. Average age: 36.79 (11.58) 3. 68.4% female	1. n: 68 (healthy controls) 2. Average age: 41.0 (13.11) 3. 61.8% female 1. n: 31 (gambling dx) 2. Average age:: 46.35 (14.52) 3. 45.2% female	-ve, +ve	UPPS-P; α: *nd*	1. YMRS; α: *nd* 2. HAMD; α: *nd*	Negative Urgency 1. BD: 2.57 (0.65) 2. HC: 1.83 (0.37) 3. Gambling Dx: 2.61 (0.58) 4. *F* = 36.11, *p* <.001; η_p_^2^ = 0.38 5. BD > HC: *p <*.01 6. BD = Gambling Dx: *ns*	*nd*
								Positive Urgency 1. BP: 2.62 (0.66) 2. HC: 1.72 (0.29) 2. Gambling Dx: 2.18 (0.49) 3. *F*(2,115) = 37.79; *p* <.001, η_p_^2^ = 0.40 5. BP > HC: *p* <.01 6. BP > Gambling Dx: *p* =.05	
Johnson et al. (2019)	United States	SCID	1. n:24 (BP I only) 2. Average age: 37.04 (9.87) 3. 50.0% female	1. n: 24 (matched controls) 2. Average age: 33.92 (12.15) 3. 45.8% female	+ve	PUM; α: 0.97	MHRSD: α: 0.92 BRMS: α: 0.94	1. BP I: 33.35 (10.15) 2. HC: 18.26 (5.42) 3. *p* <.001; *d* = 1.85	*nd*

*Note* HC denotes Healthy Controls; BP I denotes bipolar I disorder; BP II denotes bipolar II disorder; SCID denotes the use of Structured Clinical Interview for Diagnostic and Statistical Manual of Mental Disorders 4th version (DSM-IV); SCID-5 denotes the use of SCID DSM-5; +ve and -ve valence refer to positive urgency or negative urgency respectively; UPPS is Urgency, Premeditation (lack of), Perseverance (lack of), Sensation Seeking, Positive Urgency, Impulsive Behavior Scale by Whitelam et al., 2001; UPPS-P is the UPPS scale combined with positive urgency scale; PUM is the Positive Urgency Measure by Cyders et al. 2007; BRMS is the Bech Rafaelsen Mania Scale by Bech et al., 1979; MHRSD is the Modified Hamilton Rating Scale for Depression by Miller et al. 1985; YMRS is the Young Mania Rating Scale by Young et al., 1978; HAMD is the Hamilton Depression Rating Scale by Hamilton, 1960; MADRS is the Montgomery–Åsberg depression rating scale; Avg denotes Average; Hx denotes history; Dx denotes disorder; n denotes sample size; d denotes Cohen’s d effect size; α denotes Cronbach alpha; Kwapil (2013) did not report between-group differences or association with mood severity and hence removed

### Bipolar disorder vs. healthy controls

Five out of ten studies compared emotional urgency scores between bipolar disorders and healthy controls [54, 59–62]. In these studies, healthy controls were commonly individuals who were assessed in structured interviews to have no psychiatric illness; only one study had used matched controls [60]. Overall, the results overwhelmingly suggest that individuals with bipolar disorders consistently report substantially high tendencies of impulsivity during intense positive and negative mood. All five studies found significant between-group differences of negative urgency with large effect sizes (partial eta-square = 0.23 [61]; calculated Cohen’s *d* = 2.20 [59]; calculated Cohen’s *d* = 1.40 [62]), and positive urgency (partial eta-square = 0.32 [61]; calculated Cohen’s *d* = 1.82 [54]; calculated Cohen’s *d* = 1.86 [60]; and calculated Cohen’s *d* = 1.77 [62]).

### Bipolar disorders vs. other comparison groups

Four out of ten studies compared differences in emotional urgency scores between bipolar disorder and various other groups—each study’s comparison group comprising of individuals with other psychiatric illnesses or clinical attributes [59, 62–64]. Overall, negative urgency was not endorsed significantly differently within mood disorders. One study reported no significant group differences in negative urgency between bipolar disorder and depressive disorder [63]. Additionally, no group differences were reported between individuals with bipolar disorders with severe depressive symptoms than those without [59]. None of the studies reviewed had investigated between-group differences in positive urgency.

Individuals with borderline personality disorder endorsed significantly greater negative urgency than individuals with bipolar disorders [59]. Another study found between-group differences that were borderline significant in positive urgency between gambling disorder (*n* = 31) and bipolar disorder (*n* = 19, *p* =.05; [62]; Individuals with gambling disorder endorsed lower levels of positive urgency than individuals with bipolar disorder. Within the context of suicidality, individuals with bipolar disorders and with a history of attempt (*n* = 12) did not differ significantly in negative or positive urgency scores than those without any history of attempt (*n* = 18, *p* =.06 to.07; [64]) but alike the aforementioned study [62], the lack of significance (or borderline significance) could be due to a lack of statistical power.

### Association between emotional urgency and mood severity

None of the three studies that had examined the link between depression severity and negative urgency found support for its association [55, 59, 61]. Positive urgency too was not significantly associated with mania/hypomania severity in two studies [61, 65]. On the other hand, one study found a positive correlation between positive urgency and depression severity [54] which corroborated findings of another study which reported that higher positive urgency was associated with higher frequency of Major Depressive Disorder (MDD) episodes [55]. Though it had been noted in a previous study that emotional urgency was positively associated with having a history of bipolar disorder, i.e., *r* =.30 and.50 for negative and positive urgency respectively [61], prevailing empirical evidence, on the other hand, shows a lack of a linear association with depressive/mania/hypomania severity.

### Association with psychiatric comorbidity and maladaptive behaviours

A total of four studies reported the association between emotional urgency and psychiatric comorbid conditions or maladaptive behaviours [54, 55, 61, 63]. Negative urgency was positively related to anxiety, impulse control (e.g., kleptomania, pathological gambling), and substance use disorders, with *b* ranging from 0.20 to 0.34 in one study [61], and with *r* ranging from .27 to .37 in another [55]. Two out of three studies [54, 55, 61] that had examined the association between positive urgency and comorbid conditions found a significant positive correlation with substance use disorders only, ranging from *r* = .22 to .34 [55, 65].

Three distinct types of maladaptive behaviours—problem gambling, suicidality, and self-harm behaviours—were investigated separately in two studies [55, 63]. Negative urgency was not associated with self-report problem gambling [63]. Positive and negative urgency were independently and positively associated with suicide ideation, *r* = .20 and .50 respectively. Positive urgency was positively associated with suicide attempt and self-harm, *r* = .20 respectively [55]. However, after accounting for sociodemographic and clinical covariates, only negative urgency had a significant positive influence on self-harm and suicidality [55].

### Association with personality, aggression, trauma

Four studies investigated trait-like constructs of temperament, aggression, and childhood trauma history [54, 55, 59, 66] among those with bipolar disorder. Greater scores in negative urgency was associated with higher neuroticism (*b* = 0.30), and extraversion (*b* = 0.160) traits, but was associated with lower agreeableness (*b* = − 0.32) and conscientiousness (*b* = − 0.22888) aspects of personality [66]. The study further noted that negative urgency predicted cyclothymic/irritable temperament—a combined characteristic of mood and negative affect reactivity during negative life episodes (*b* = 0.40; [66]). Next, negative urgency was highly associated with all aspects of childhood trauma, such as emotional abuse, physical and emotional neglect, *r* estimates with a medium-high effect, ranging from 0.48 to 0.69. However, there was no significant relationship between trauma history and emotional urgency [55]. The strongest association was found for emotional neglect [59]. In the context of aggression and dominance, positive urgency was significantly associated with anger, hostility, physical and verbal aggression, *r* ranging from 0.38 to 0.51 [54].

### Association with quality of life and functioning

Quality of life and functioning were operationalized by two widely used validated scales: the quality of life in bipolar disorder (QOL-BD) scale by Michalak et al. (2010) and the global assessment of functioning (GAF) scale, respectively. Positive urgency was negatively correlated with quality of life (*r* = -.50; [56]), negatively associated with overall functioning, (β = -0.40 to -0.45), and had accounted for a significant amount of variance (14–24%) of overall quality of life or functioning scores [56, 61].

## Discussion

A scoping review was conducted to exploratorily determine the extent of available evidence of the clinically significance of emotional urgency in BD. Overall, there is support for the clinical relevancy of emotional urgency in the extant literature. Across studies, individuals with bipolar disorders consistently endorsed higher levels of emotional urgency than healthy individuals. There is moreover a high percentage of statistical variance of quality of life and functioning scores that are explained by emotional urgency, which supports its relevance to clinical recovery. There is, however, a lack of consistent evidence for the association between negative urgency and mania or depression severity. Finally, existing data suggests that emotional urgency is not endorsed any differently across mood disorders, providing preliminary support for its transdiagnostic nature.

Empirical evidence based on community data found significant associations between emotional urgency and mania or depression risk/severity [67, 68], but this was not strongly evident in clinical samples. This could imply that, like other non-emotion-based trait pathways of impulsivity, heightened emotional urgency is a stable trait of bipolar disorder regardless of illness phases or mood state. However, important limitations have to be noted and addressed, such as the lack of sufficiently powered studies to detect associations [59, 61] and the lack of an account of disorder heterogeneity. Within mania, the types of mood experience can vary greatly, ranging from euphoria to dysphoric emotions [69]. Certain manic features, such as irritability, may be more associated with negative urgency than other features [66]. Similarly, the predominant polarity of the illness (e.g., individuals who experience primarily manic/hypomanic or depressive episodes or no predominant polarity); [70–72] have shown to influence impulsivity levels. Only two out of four studies had accounted for illness phases [54, 61] and no studies reviewed had considered the role of clinical relevant moderators, such as predominant polarity or mixed mood states [73]. Future research could parse emotional urgency by mood features rather than in broad general dimensions of hedonic mood and severity to further delineate the role of emotional urgency.

The relationship between emotional urgency and maladaptive behaviours varied widely between studies. In general, this review found preliminary support for a positive association with aggression, and hostility constructs, childhood trauma, and suicide ideation which corroborates past research [24, 34, 65, 74, 75]. Associations with medium to large effect sizes were found for anger and hostility constructs of aggression, major forms of childhood trauma, and suicide ideation (see Tables 1 and 2 for a clearer summary). Positive but not negative urgency was associated with self-harm and suicide attempt [47, 76, 77] which was an unexpected finding. This could be explained by the understanding that different facets of suicidality may be differentially linked to impulsivity [78, 79]. In certain contexts, emotional urgency may act as an amplifier [78, 79] or be moderated by other constructs of suicidality [69].

### Limitations

Several important limitations must be considered before concrete conclusions can be made. Most crucially, as the aim of this scoping review was to descriptively summarize results of existing studies, it does not allow for drawing conclusions beyond integrated findings. Secondly, due to the limitations of a scoping review, the quality of studies remains to be assessed by future systematic reviews. Thirdly, most studies were designed to detect group differences but not associations— the latter investigation was often not part of the main study design focus, and thus, the outcomes of this review were significantly hampered by the lack of adequately powered studies. As most studies reviewed had investigated negative or positive urgency alone (i.e., studies that had utilized the UPPS or PUM, but not the updated version of UPPS-P, which contained both negative and positive urgency dimensions), this review is unable to ascertain which dimension played a more significant role in bipolar disorders. As a result of these limitations, a greater volume of basic research using diverse cultural samples is needed to validate and generalize the findings of this review and expand knowledge on emotion-based impulsivity in bipolar disorders (See Table 2).

**Table 2:** The correlates of emotional urgency

Authors	Urgency Valence	Clinical/Psychosocial correlate or maladaptive behaviour	Corresponding measure	r or b coefficient	*p* value
Quilty et al. (2010)	-ve	Problem gambling pathology severity	a. Canadian Problem Gambling Questionnaire (CPGI) by Ferris & Wynne 2001 1. 31 items 2. Scores > 8 indicates severe risk of problem gambling 3. α: 0.95 b. South Oaks Gambling Screen (SOGS) by Lesieur & Blume, 1974 1. 16 items 2. Scores > 5 indicative of problem gambling 3. α: 0.94 c. Problem Gambling Modification of the Yale-Brown Obsessive Compulsive Scale (PG-YBOCS) by Pallanti et al. 2005 1. 10 items; Subscales: Urges, Behaviours 2. Scores > 24 show severe gambling difficulty 3. α: 0.9 (urges), 0.89 (behaviours)	a. *r* =.08 b. *r* =.13 c. *r* =.11 (total); *r* =.10 (urges); *r* =.12 (behaviour)	all ns
Victor et al. (2011)	+ve	Quality of life	Quality of Life in Bipolar Disorder (QoL-BD) by Michalak et al., 2010	a. *r* = -.52 hierarchical linear regression analysis showed that Positive Urgency explained a significant amount of variance of overall QOL (criterion variable) b. Total model (step 1:site; step 2: comorbid diagnoses; step 3: Barratt’s Impulsivity Score and PUM): F(3, 63) = 7.18, *p* <.001, *R*^2^ = 36.3%; *b* =-0.40, *t* = -3.7 c. 3rd Block (positive urgency): F(1,63) = 13.74, Δ*R*^2^ = 0.14; only positive urgency significantly predicted QOL.	a. *p* <.001 b. *p* <.0005 c. *p* < 0.0005
Kwapil et al. (2013)	-ve	Personality and temperament	a-e. NEO Five-Factor Inventory (NEO-PI-3) by Costa and McCrae (1992); α: 0.48 (Openness-3) to 0.82 (Neuroticism-3) f-g. Temperament Evaluation of Memphis, Pisa, Paris, and San Diego—Autoquestionnaire (TEMPS-A) by Akiskal et al., 2005a; Cyclothymic/Irritable temperament; α (combined): 0.86 e. Hypomanic Personality Scale (HPS) by Eckblad & Chapman 1986; α: *nd*;	Negative Urgency is the criterion variable a. Neuroticism (step 1): *b* = 0.336 b. Extraversion (step 1): *b* = 0.160 c. Openness (step 1): *b* = − 0.017 d. Agreeableness (step 1): *b* = − 0.32 e. Conscientiousness (step 1): *b* = − 0.218 f. Hyperthymic temperament (step 2): *b* = − 0.063 g. Cyclothymic/Irritable temperament (step 2): *b* = 0.398 e. HPS: *nd*	a. *p* <.001 b. *p* <.05 c. ns d. *p* <.001 e. *p* <.01 f. ns g. *p* <.001 e. *nd*
Muhtadie et al. (2014)	-ve, +ve	Global Functioning Comorbid conditions: Lifetime alcohol abuse. dependence, substance abuse, dependence, and anxiety disorder	a. Global Assessment of Functioning (GAF) b. Lifetime alcohol abuse (Y/N) c. Lifetime alcohol dependence (Y/N) d. Lifetime substance abuse (Y/N) e. Lifetime substance dependence (Y/N) f. Anxiety disorder (Y/N) g. Impulse control disorder (Y/N)	a. Positive urgency (block 3): *b* = -0.45; *t* (78) = -4.60; Final model (block 1: site; block 2: comorbid diagnosis; block 3: impulsivity measures) accounted for 42.5% of variance of GAF score, *F* (6, 75) = 5.27; Positive urgency (final 3rd block) accounted for 24.2% of variance of GAF score; Negative urgency did not account for significant variance of GAF. Did comorbid conditions predict Positive Urgency b. *nd* c. *nd* d. *nd* e. *b = 0.09* f. *nd* g. *b =* 0.13 comorbidity Δ*R*^2^ = 0.15 *p* <.001 Did comorbid conditions predict Negative Urgency b. *nd* c. *nd* d. *nd* e. *b* = 0.14 f. *b* = 0.24 g. *b* = 0.20 comorbidity Δ*R*^2^ = 0.30 *p* <.001	g. *p* <.001 *b. p* <.001 Positive Urgency b. *ns* c. *ns* d. *ns* e. *ns* f. *ns* g. *ns* Negative Urgency b. *ns* c. *ns* d. *ns* e. *p* <.05 f. *p* <.05 g. *p* <.05
Bøen et al. (2015)	-ve	Childhood Trauma	Short Childhood Trauma Questionnaire (CTQ) by Bernsteinetal.,2003	a. CTQ total: *r* =.68 b. emotional abuse: *r* =.65 c. emotional neglect: *r* =.69 d. physical neglect: *r* =.48	a. *p* =.001 b. *p* =.003 c. *p* =.001 d. *p* =.04
Johnson et al. (2016)	+ve	Aggression	Aggression Questionnaire (AQ) Short version by Buss and Perry, 1992; the 4 subscales are: a. verbal aggression (α = 0.82) b. physical aggression (α = 0.74) c. Anger (α = 0.77) d. Hostility (α = 0.80) e. Sense of Power Scale (SPS) by Anderson et al., 2012; α = 0.87 f. Personality Research Form Dominance Motivation (PRF-Dom) by Jacson, 1999; α = 0.81 g. Trauma history screen by Carlson et al., 2011; α: *nd*	a. verbal aggression: *r* =.38 b. physical aggression: *r* =.38 c. anger: *r* =.51 d. hostility: *r* =.47 e. SPS: *r* = -.34 f. PRF-Dom: *r* = -.08 g. Trauma Hx: *r* =.37 did PUM predict criterion variables a-g? a. *b* = 0.30, *p* =.03; Overall model fit: F (2, 51) = 5.00, *p* =.01, R^2^ = 16% (positive urgency was the sole significant contributor) b. *nd* c. *b* = 0.41, *p* =.001; Overall model fit: F (3, 54) = 7.71, *p* <.001, R^2^ = 26% (positive urgency was the sole significant contributor) d. *b* = 0.30, *p* =.02; Overall model fit: *F* (4, 52) = 8.81, *p* <.001, R^2^ = 40% (positive urgency and perceived power score were significant contributors)	a. *p* <.01 b. *p* <.01 c. *p* <.001 d. *p* <.001 e. *p* <.01 f. *ns* g. *p* <.01
Johnson et al. (2017)	-ve, +ve	Trauma	a. Trauma history screen by Carlson et al., 2011 b. History of Self-harm (Y/N) c. History of suicidal ideation (Y/N) d. History of suicide attempt (Y/N) e. Lifetime substance diagnoses (Y/N) f. Lifetime anxiety diagnoses (Y/N)	Positive urgency (*n =* 133) a. *r* =.22 b. *r* =.20 c. *r* =.20 d. *r* =.20 e. lifetime substance: *r =*.22 f. lifetime anxiety: *r* =.14 Did positive urgency predict criterion variables b-d? b. *b* = 0.14, t = 1.62, *p* =.11, semi-partial *r* =.14; overall model explained 15.9% of the variance of self-harm, *F*(3, 121) = 7.43, *p* <.001. c. *b* = 0.10, t = 1.16, *p* =.25, semi-partial *r* =.09; overall model explained 19.7% of the variance of suicidal ideation, *F*(4,130) = 7.75, *p* <.001 d. *b* = 0.15, t = 1.68, *p* =.10, semi-partial *r* =.14; overall model explained 8.3% of the variance of suicide attempts, *F*(2, 131) = 5.86, *p* =.004 Negative urgency (*n* = 75) a. *nd*; b. *r* =.18 c. *r* =.52 d. *r* =.2 e. lifetime substance: *r =*.27 f. lifetime anxiety: *r* =.37 Did negative urgency predict criterion variables c-d? c. *b* = 0.41, *p* =.001; overall model (block 1: comorbid conditions, MDD frequency, PUM; block 2: negative urgency) explained 19.7% of the variance of suicidal ideation, *F*(4, 130) = 7.75, *p* <.001; block 2 (negative urgency): *F* (5, 73) = 6.98, *p* <.001, *r*^2^ = 0.34	positive urgency a. *ns* b. *p* <.05 c. *p* <.05 d. *p* <.05 e. *p* <.05 f. *ns* negative urgency a. *ns* b. *ns* c. *p* <.001 d. *ns* e. *p* <.05 f. *p* <.01

### Future directions, clinical implications, and conclusion

Emotional urgency is a promising concept of trait impulsivity due to its profoundly large clinical significance in psychopathology. Similarly, large effect sizes were found in this scoping review extending support for future research in bipolar disorder. At this juncture, more basic science studies with adequate statistical power must be conducted to thoroughly elucidate its role in mood dysfunction. While it is premature to draw any real clinical implications, a consolidation of existing work can inform future directions pertaining to its role in bipolar disorders and disorders of mood dysfunction in general.

One important characteristic of trait concepts is its inherent nature, and thus, incorporating emotional urgency into clinical interventions may pose a challenge due to its resistance to change. However, recent work does suggest that negative urgency can impede therapeutic success if left unaddressed [80–82], and further evidence does show that negative urgency can be significantly reduced by existing psychological interventions, such as dialectic behavioural therapy and cognitive behavioural therapy [83, 84].

Finally, the gathered evidence suggests that there may be great value in investigating the role of emotional urgency in the context of a theoretical framework—to elucidate its role as an indirect or conditional variable within conceptual models. Parsing emotional urgency and mood states in the context of cognition and emotional dysregulation in mood disorders [28, 32, 85–87] for instance, could further ascertain the role of emotional urgency in complex maladaptive behaviours.

## Data Availability

No datasets were generated or analysed during the current study.

## Abbreviations

BIS: Barratt’s Impulsivity Scale
BD: Bipolar Disorder(s)
DSM: Diagnostic Statistical Manual of Mental Disorders
GAF: the Global Assessment of Functioning scale
MDD: Major Depressive Disorder
NSSI: Non-suicidal Self-Injurious behaviours
PUM: Positive Urgency Measure
PRISMA: Preferred Reporting Items for Systematic Reviews and Meta-Analyses
PRISMA-ScR: Preferred Reporting Items for Systematic Reviews and Meta-Analyses (PRISMA) checklists and guidelines for Scoping Reviews
PROSPERO: the International Prospective Register of Systematic Reviews
QOL-BD: the Quality of Life in Bipolar Disorder scale
UPPS-P: Urgency, Perseverance, Premeditation, Sensation Seeking, and Positive Urgency
